# The what and where of primate field research may be failing primate conservation

**DOI:** 10.1002/evan.21790

**Published:** 2019-07-25

**Authors:** Michelle Bezanson, Allison McNamara

**Affiliations:** ^1^ Department of Anthropology Santa Clara University Santa Clara California; ^2^ Department of Anthropology The University of Texas at Austin Austin Texas

**Keywords:** conservation, ethics, fieldwork, nonhuman primates, primatologists, publications

## Abstract

With approximately 30% of nonhuman primate species listed as critically endangered, the window of opportunity to conserve primates is closing fast. In this article, we focus on the degree to which publications in field primatology are biased in favor of particular taxa and field sites. We examined more than 29,000 peer‐reviewed articles and identified 876 field visits to 349 field sites. We found a highly clumped distribution by site and species. We also examined publication ethical statements and the extent to which they acknowledged local human communities (<5%). Due to a lack of consistency across publications, we provide recommendations for improving ethical statements and for evaluating research impact. Given the plight of primate biodiversity, these results suggest broader coverage of primate species and geographies, as well as more attention to the local human communities whose support is necessary if the intent is to have primate species in the wild in the 22nd century.

## 1 INTRODUCTION

Much on‐the‐ground conservation is aimed at protecting particular species—often charismatic species such as orangutans, rhinos, tigers, bald eagles, and so forth. Living nonhuman primates (primates hereafter) stand out as a group of singularly charismatic species that command attention worldwide due to their phylogenetic proximity to humans, and because images of their suffering can resonate deeply. In addition, primates are at risk throughout the world, with 60% of all primate species classified as threatened with extinction by the International Union for Conservation of Nature (IUCN).^1^ Preventing the extinction of these species requires an understanding of their biology, ecology, life history, behavior, habitat needs, evolutionary flexibility, and phenotypic plasticity. Primatologists, especially anthropological primatologists who conduct field research, are the source of most of our key insights into primate evolution, behavior, ecology, and biology that can be used to advance primate conservation.^2^, ^3^, ^4^, ^5^

Here, we focus on field research on primates to better understand how the portfolio of published research might bias, inform, and even directly impact primate conservation and anthropological frameworks. The pattern of published primate field research not only defines theoretical frameworks, but also constrains future conservation outcomes. It will become increasingly challenging to conserve primate populations whose behavior and ecology are unknown. Additionally, the interactions of primatologists with local communities could constrain conservation success—if researchers are viewed as exploitive, and if they do not acknowledge the help of local communities, all the knowledge in the world about primates could be for naught.

To understand primatological field research patterns, we examined 5 years of published primate field research (over 29,000 articles), and asked: what issues does a quantitative description of that record of primate field research raise, with respect to primate conservation? While our focus is primates, this thread of inquiry could be useful for any taxonomically defined research. In the end, it is essential to appreciate that conservation is about averting the extinction of species, and scientists who study the ecology, biology, and behavior of species occupy a special position in conservation. Primatologists represent and provide the depth of knowledge that gives us our best chance for saving primate species. We therefore examine how a nonrandom distribution of publications from field sites that primatologists visit, and the species they choose to study, may constrain our understanding of opportunities for primate conservation. We also examine the extent to which primatologists acknowledge local communities. While every primatologist need not be a conservationist, as a scientific field it would be irresponsible if primatology did not self‐examine its activities, and ask how primate field research might better serve primate conservation.

Biological field sites and research stations that primatologists visit play a critical role in longitudinal ecological monitoring, innovative research, and conservation. They have the potential to contribute to local human community infrastructure and sustainable development.^6^, ^7^, ^8^, ^9^, ^10^, ^11^, ^12^ Field sites/stations allow understanding of natural history, evolution, and behavior, while inspiring students, members of local communities, and global citizens. Many researchers cite a field experience as inspiration for their current work.^13^, ^14^ Researchers argue that there are many benefits to research at long‐term field sites including improving the understanding of primate behavioral variability, understanding the effects of climate change, and measuring responses to selective logging, as well as establishing consistent funding and benefits to local human communities.^10^^,^ 15, 16 In addition, working at long‐term sites can be attractive due to infrastructure, logistical support, baseline data to build on, and a community of researchers during fieldwork. These advantages, however, may bring with them a tradeoff of such spatially and taxonomically biased (clustered) research, that the broader picture of primates, their flexibility, and threats to conservation are neglected. While the importance of field stations is recognized, very little systematic study of field station activities, data management strategies, impacts on ecosystems, impacts on local communities, and impacts on primate conservation has been published to date.^17^, ^18^, ^19^, ^20^, ^21^, ^22^

During the last two decades, primatological research has moved toward increased research specialization, the integration of biological and behavioral measures, technological innovation for examining primates remotely, and the examination of human/nonhuman primate interactions.^23^, ^24^, ^25^, ^26^, ^27^ The support of local people living adjacent to primate habitats has been key to conservation success and many researchers engage in conservation initiatives while conducting research.^28^, ^29^ Some journals require ethical statements that include details of Human Subjects/IRB (Institutional Review Board) permissions, IACUC (Institutional Animal Care and Use Committee) permissions, and legal compliance. At this time, ethical statements do not require details of local community involvement or impact. For example, is support for the local community consistent? Does the local community have access to the area? What is the history of land use in the area? How do scientific activities at the field site affect the local human community and landscapes? Ethical implications that may be included in ethical statements include describing conservation outcomes, detailing community support, and assessing the impacts of research on neighboring human communities. It is imperative that field researchers pay careful attention to the ethical implications of their work at all stages of the research process from conception to publication, and that these implications be included in publications so that we may track the progress of our efforts.

## 2 WHAT CAN WE LEARN FROM THE PRIMATE LITERATURE?

### 2.1 Quantifying where primatologists are working, and the species they are working on

We sampled the primate literature over a recent 5‐year period: 2011–2015. We restricted our search to issues of the *American Journal of Primatology*, *International Journal of Primatology*, *Primates*, *American Journal of Physical Anthropology*, *Journal of Human Evolution*, *Nature*, *Science*, and *Proceedings of the National Academy of Sciences*. We focused on top‐ranking anthropological primatology and broader science journals because these publications receive great attention from the scientific community and have immense impact on our collective understanding of the behavior and biology of primates.

Our goal was to identify where primatologists are working, and the species they are working on, based on the primate literature. We follow Estrada et al.'s^1^ classification (701 extant taxa belonging to 504 species, 79 genera) and the four region definitions of North America/South America, Mainland Africa, Madagascar, and Asia. We ask the following questions: (1) Where do primatologists who publish in these journals work? (2) What taxa are they studying? (3) To what extent do these research publications explicitly include/address the ethical implications of the research?

We began by reviewing each journal issue's table of contents to identify primatological field research. If the article title suggested that it involved a primatological field study (includes any primate study that required a single‐day or multiple‐day stay at a field site), we then reviewed the abstract and methods sections to verify timing, location, and the geospatial coordinates of the field site(s). If coordinates were missing from the methods, we searched using Google Earth and Google Maps to identify the location.

We sorted studies by author to remove studies that used the same data set in multiple publications. We performed a content analysis of study methods to determine site name, protected status as listed by the site or in methods (national park, reserve, privately owned, etc.), length of time in the field, species examined, habitat type, anthrome of the study site,^30^ whether the study explicitly discussed conservation implications, and the presence or absence of a variety of types of ethics statements. We focus on anthromes rather than biomes because 75% of Earth's ice‐free land has been visibly altered by human activities, anthromes incorporate human population and land use, and anthromes are more appropriate for understanding ecological impacts at field sites than traditional biome classifications.^31^ In contrast, traditional measures of primate biomes involve climate, terrain, and geology, but neglect human activities or impact in the region.

### 2.2 Global patterns of primate field research

**Figure 1:**
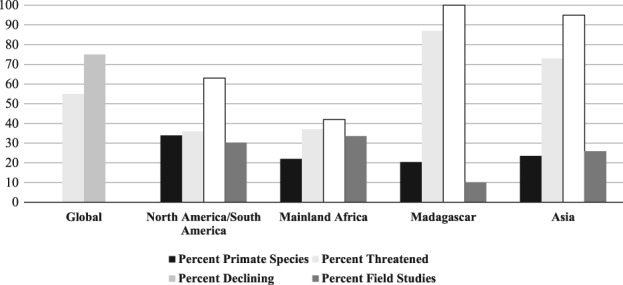
Distribution of field site visits compared to percent primate species, percent threatened species, and percent declining species (IUCN numbers^1^). The observed distribution of field visits differed significantly from the expected distribution given the number of primate species in each region (*Χ* ^2^ = 99.1, *df* = 3, *p* < .001), the number of threatened species in each region (*Χ* ^2^ = 530, *df* = 3, *p* < .001), and the number of declining species in each region (*Χ* ^2^ = 409, *df* = 3, *p* < .001)

We reviewed 29,140 article titles and identified 754 publications that included a total of 876 unique field visits to 349 sites in the North America/South America, Mainland Africa, Madagascar, and Asia. A field visit means traveling to a primate habitat location for field data, therefore, a single study may involve multiple site visits. More than half (64.7%) of the research articles listed geospatial coordinates. We were able to find the majority of the remaining field site locations (33%) in articles using Google Earth in combination with Google Maps (2017). We produced maps using ArcGIS and used the Ellis and Ramankutty^31^ anthrome layer to show the proximity of field sites to 19 land use categories.^32^ We then reduced the 19 anthrome categories into five broad categories: Urban (Urban and Mixed Settlements), Village (all “Village” categories), Cropland (all “Cropland” categories), Rangeland (all “Rangeland” categories), Woodland/Forest (the remaining “woodland” and “treeless” categories).^30^ We compared the observed distribution of field site visits to the distribution expected given the number of primate species and number of endangered species in each region, following methods in recent conservation publications.^1^^,^ 33 Goodness‐of‐fit chi‐square values were calculated in R v.3.4.3.^34^ From 2011–2015, primatologists published research in Mainland Africa most often (45.6% of field studies), followed by the North America/South America (29.2%), Asia (25.1%), and Madagascar (9.9%). We found that the observed distribution of field visits differed significantly from the expected distribution given the number of primate species in each region, the number of threatened species in each region, and the number of declining species in each region (Figure 1).

The majority of primatological fieldwork took place in forested regions (80.0%), followed by villages (8.5%), rangelands (4.2%), urban areas (3.6%), and croplands (3.5%). The span of time represented by the published data at the field sites averaged 17.2 months (range: 1 day to 388 months, *SD*: 27.5 months). The majority of researchers published studies based on data collection for 12 months or fewer in the field (51.5%) while 37.7% of the studies totaled 13 months or more of data collection in the field. Anecdotes or studies of fewer than 23 days totaled 3.3%. Many researchers did not provide dates of research (13.8%).

### 2.3 Primate field sites within each region

**Figure 2:**
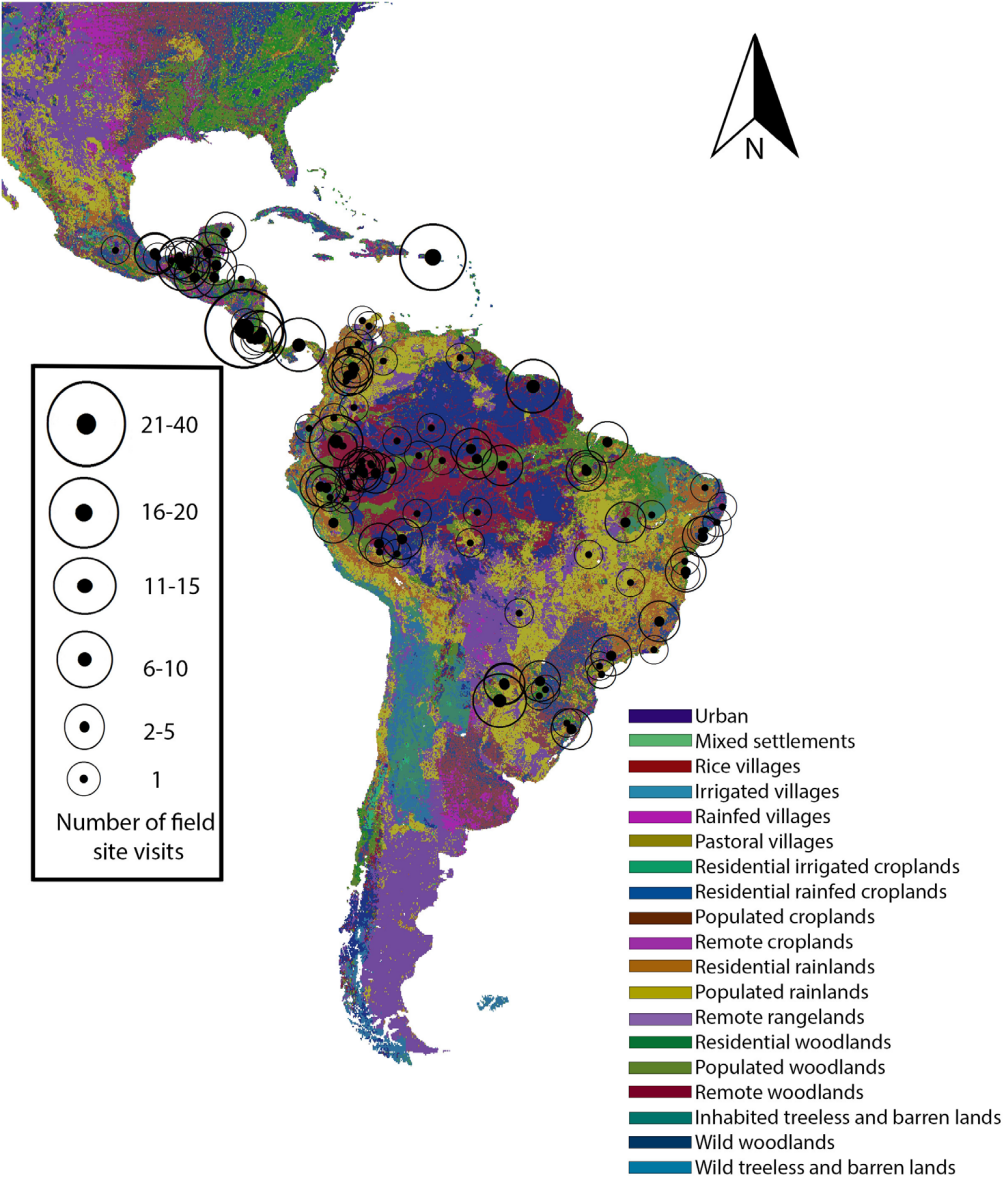
Published primatology visits to field sites, 2011–2015: North/South America. ArcGis Anthrome layer^31^, ^32^ [Color figure can be viewed at wileyonlinelibrary.com]

The publications indicated that these primatologists worked in protected areas most often with 73.3% of field visits taking place in national parks/protected areas and 26.7% of field visits to privately owned field sites, unprotected areas, urban areas, or sites of unknown protected status. There were 256 field site visits to North America/South America (Figure 2), where Santa Rosa National Park (*N* = 17), Cayo Santiago (14), Yasuní National Park (10), Isla Brasiliaera (8), Palenque National Park (7), and Barro Colorado National Park (6) experienced the highest researcher traffic (total 62/256 = 24.2%). The following North American/South American primate habitat countries were not represented in the literature searched in this study: French Guiana, Guatemala, El Salvador, Paraguay, and Trinidad and Tobago.

**Figure 3:**
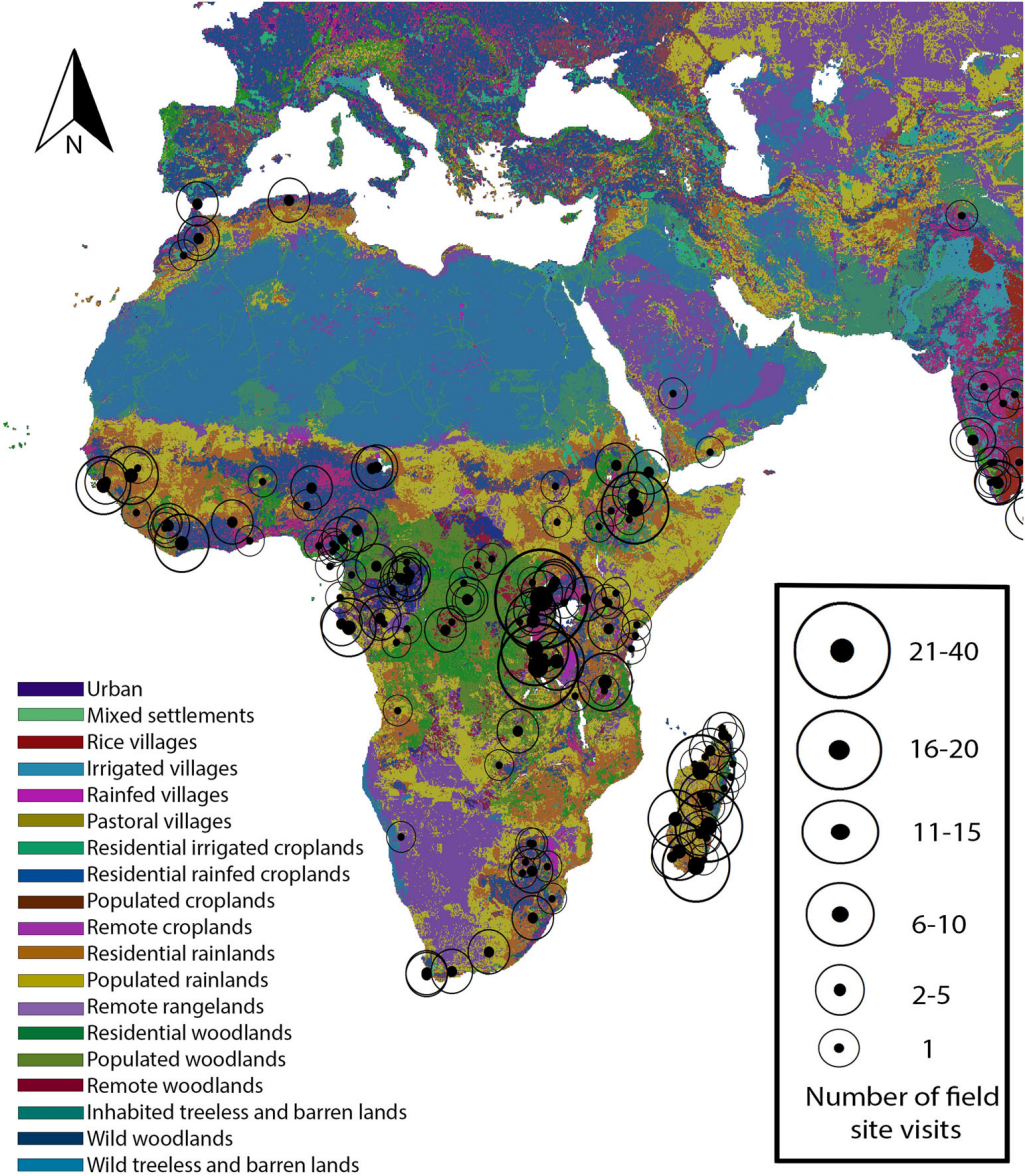
Published primatology visits to field sites, 2011–2015: Continental Africa and Madagascar. ArcGis Anthrome layer^31^, ^32^ [Color figure can be viewed at wileyonlinelibrary.com]

In Mainland Africa (Figure 3), publications represented 313 field visits with Kibale National Park (40), Mahale Mountain National Park (16), Awash National Park (14), Gombe Stream National Park (14), and Taï National Park (8) experiencing high researcher traffic (total 92/313 = 29.4%). The following primate habitat countries from Mainland Africa were not represented in the literature searched in this study: Benin, Botswana, Burundi, Chad, Djibouti, Eritrea, The Gambia, Guinea, Lesotho, Malawi, Mali, Mauritania, Mozambique, Niger, Somalia, Sudan, Swaziland, Togo, and Zimbabwe.

There were 87 visits to Madagascar (Figure 3) represented in the publications sampled with the highest researcher traffic at Ranomafana National Park (13), Berenty Private Reserve (12), Kirindy Forest National Park (10), and Ankarafantsika National Park (8) (total 43/87 = 49.4%).

**Figure 4:**
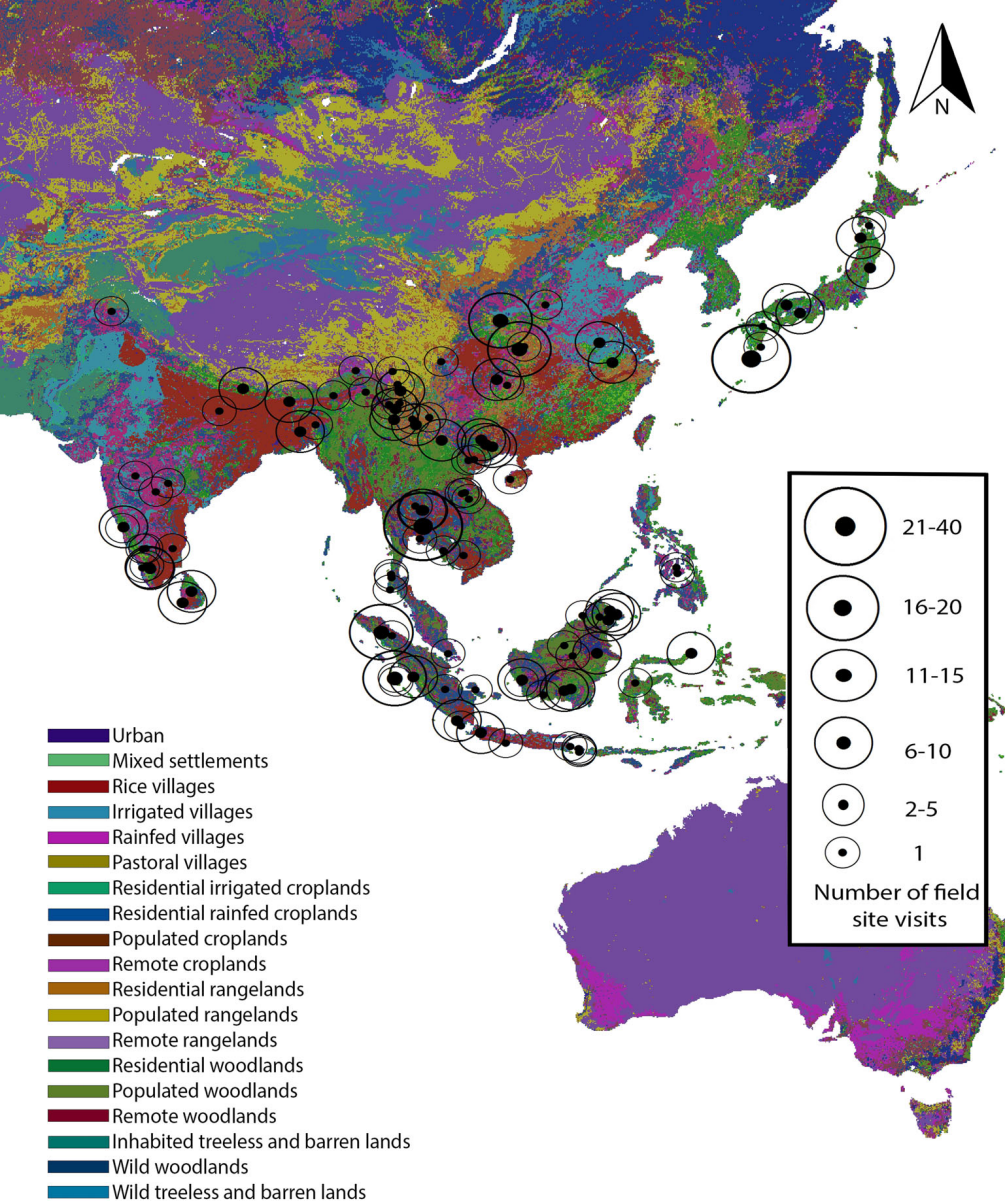
Published primatology visits to field sites, 2011–2015: Asia. ArcGis Anthrome layer^31^, ^32^ [Color figure can be viewed at wileyonlinelibrary.com]

In Asia (Figure 4), there were 220 site visits represented in the publications with Khao Yai National Park (17) experiencing the heaviest researcher traffic followed by Yakushima Island National Park (11), Zhouzhi National Nature Reserve (9), and Sabangau Forest (8) (total 51/220 = 23.2%). The following Asian primate habitat countries were not represented in the literature searched in this study: Afghanistan, Brunei, Bhutan, Singapore, Timor‐Leste, Taiwan, and Yemen.

### 2.4 Species diversity of primate field sites

**Figure 5:**
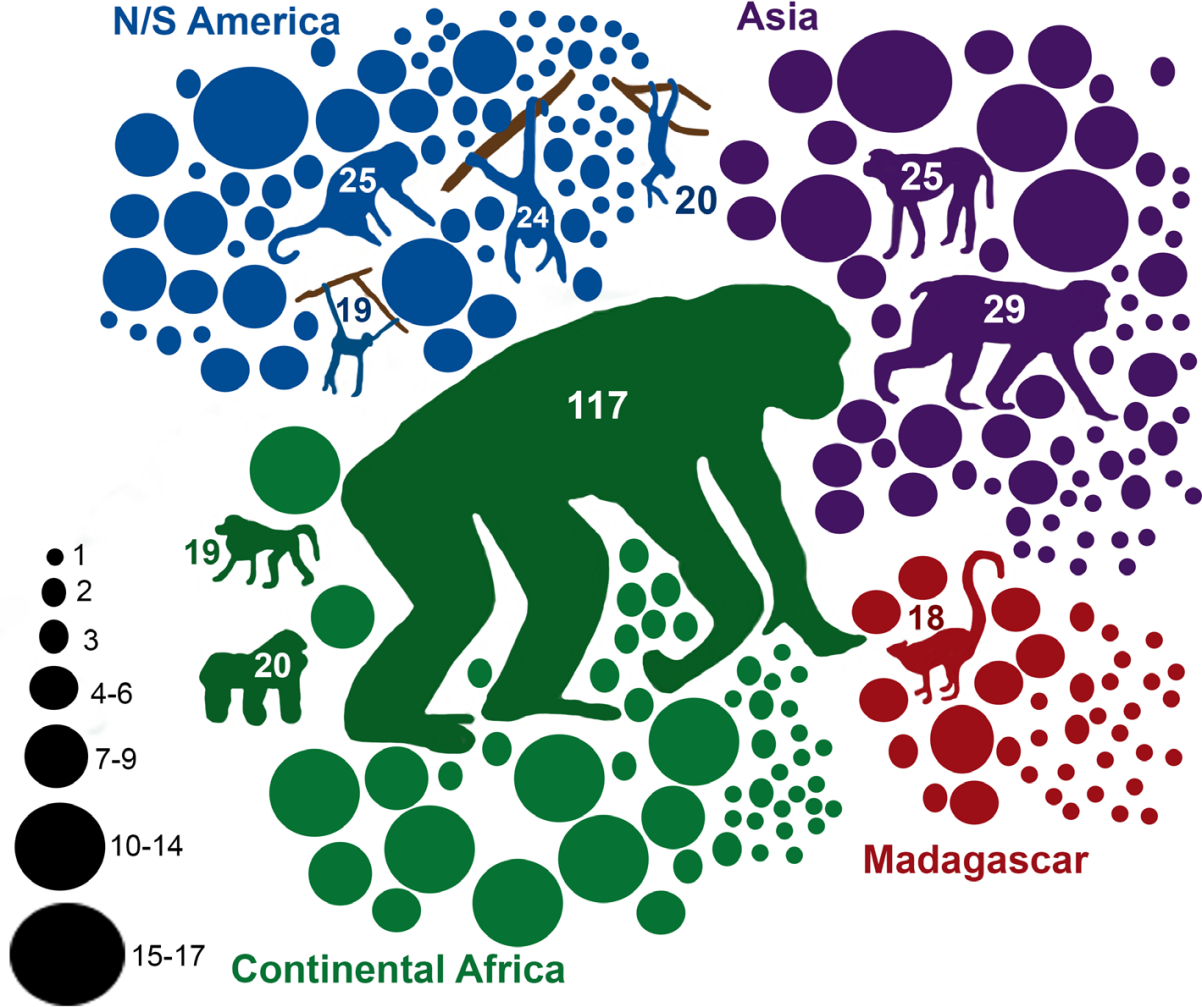
Species represented in field primatology publications 2011–2015. Top 10 species represented by species‐specific silhouettes labeled with number of field visits: *Pan troglodytes*, *Macaca fuscata*, *Macaca mullata*, *Alouatta palliata*, *Alouatta pigra*, *Gorilla gorilla*, *Cebus capucinus*, *Papio hamadryas*, *Ateles geoffroyi*, *Lemur catta*. All other species are represented by dots, the size of which reflects the number of field visits. In all, 240 species were examined in 876 field visits

Primatologists published their work on 240 (47.6%) of the 504 currently recognized primate species during the 5‐year period (Figure 5). The published work was largely focused on chimpanzees (*Pan*), macaques (*Macaca*), howlers (*Alouatta*), and capuchins (*Cebus/Sapajus*). Specifically, primatologists focused much of their fieldwork on *Pan troglodytes* (13.3%) followed by *Macaca fuscata* (3.3%), *Macaca mulatta* (2.8%), *Alouatta palliata* (2.8%), *Alouatta pigra* (2.7%), and *Gorilla gorilla* (2.2%). Overall, 30% of primates are currently considered critically endangered (CR), data deficient (DD), or no evaluation (NE) exists, but only 18% published papers concerned these IUCN categories of species.

### 2.5 Primate field studies focusing on conservation

The literature represented in this study was primarily (82.4%) focused on topics other than conservation. Only 17.6% of the publications were conservation‐focused or provided conservation implications. The majority of conservation‐focused publications addressed anthropogenic influences on habitat (47.7%), population status/density (20.5%), and ethnoprimatological approaches to human/wildlife interactions/conflict (13.6%). The remaining conservation studies focused on remote/noninvasive methods (4.5%), population health (4.5%), genetic diversity (3.8%), reintroduction (3.0%), and the effects of habituation (2.4%).

### 2.6 Ethics statements associated with primate field studies

The majority of published field studies mentioned governmental agency permission, animal care and use, and/or acknowledged assistants and local community members. Almost half (48.2%) of studies provided an explicit ethics statement in the methods or acknowledgments section. By explicit, we mean that the section was either labeled as such or appeared in a stand‐alone paragraph within the section. In 39.6% of studies, permission, or animal care and use was mentioned briefly in the acknowledgments or methods. We found no evidence of permissions, following legal requirements, or animal care and use in 12.2% of the publications. More than half of the publications (53.4%) thanked field assistants or project staff by name. Very few publications thanked local community members (4.4%) and 31.2% of the publications made no mention of field assistants, local community members, or other support while in the field.

## 3 MOVING FORWARD

Field primatologists are publishing on a small portion of taxa and are concentrating their work at a relatively small number of research sites. There are currently 504 recognized species of primates and fewer than half of these were represented in the primate literature reviewed here.^1^ We recognize that increases in primate species numbers in recent classifications may slightly influence these numbers. For example, a species name in a particular publication may be different than the recent classification. During the time period sampled, we found that the majority of studies were performed in national parks and at long‐term established field sites. On the positive side, a high number of field site visits and a disproportionate representation of species studied at these field sites can lead to permanent field site infrastructure, long‐term community relationships, comparable results, and a better understanding of intraspecific and interspecific variation. However, focusing research to a limited number of field sites and primate taxa not only leads to a lack of information on populations, species, and regions, it also biases our understanding of primate behavioral and biological patterns and diversity.

### 3.1 Is a field site bias bad?

Yes and no. There are ethical ramifications when working at all field sites and a site's ability to attract researchers, students, tourists, and local community support can produce both positive and negative outcomes (Table 1). Arguments that support long‐term primatological field presence emphasize the ability to bring international attention to the field site, establish protected status, and improve habitat management. Other benefits include the availability of long‐term data on ecosystem health, primate habituation which facilitates observation of subjects, greater leverage in the ability to influence policy, better ability to establish relationships with local human communities, the ability to train local field assistants, and the ability to train large numbers of students.^9^^,^ 11, 12 ^,^ 16 ^,^ 35, 36, 37, 38 The scientific benefits are explicit early in field research, but it might take years to observe and assess how study systems respond in the long term, how human communities are influenced by our desire to protect threatened and endangered species, how trails influence the ecosystem, how field trash impacts the environment, and how our efforts might attract hunting or tourism. Struhsaker^11^ argued that it takes 20 years of long‐term commitment for a field site to influence government policy, establish relationships with the local community, collaborate with overseas partners, and expand the field science to larger conservation projects. Primatologists as conservation advocates tend to highlight the benefits to the human communities, however this requires long‐term assessment, ethnography, and other types of social science research to understand consequences including disproportionate benefits or alienation.^29^^,^ 39 ^,^ 40 Long‐term field sites may attract more consistent funding by integrating field schools, tourism, and other large group visits to the site. This traffic can have negative long‐term effects that may not be fully understood until they are too severe to easily correct.^17^^,^ 41, 42, 43

**Table 1:** Benefits and negative consequences of long‐term researcher presence at field sites^6^^,^ 12 ^,^ 17 ^,^ 35

	Benefits	Negative consequences
Scientific	Scientific discovery can lead to protection	Disturbs wildlife and/or ecosystem with infrastructure, trails, etc.
	Enables habituation of primate groups	Alters the behavior of study subjects and other wildlife
	Presence affords conservation value	Presence draws attention to field site for hunting, tourism, etc.
	Increases public awareness	Potential for attracting tourists and/or other exploitative users
Community	Increases local human community participation/support	Potential for excluding/alienating/negatively impacting local community
	Builds local capacity and supports local economy	Short‐term field visits may cause unequal or unpredictable economic support
	Influences policy/management, attracts government support	Potential for disease transmission
	Enhances ability to identify and report poachers	Increased poaching and hunting with increased visibility and popularity
Education/outreach	Brings tourism (increased jobs, funding)	Problematic behavior‐tourists
	Provides opportunities for educational programs (e.g., field courses and conservation education initiatives)	Problematic behavior‐students

The consequences of field work in protected areas and wildlife management are often realized after long‐term work and reflection, or through research by cultural anthropologists or ethnoprimatologists.^21^^,^ 37 ^,^ 42 ^,^ 44, 45, 46, 47, 48 Working at field sites in protected areas can have devastating effects on human communities. For example, researcher presence and implementation of new conservation restrictions may lead to policy that disallows local human communities from using the forest for which their livelihoods depend while allowing tourism to increase.^41^, ^42^^,^ 46 ^,^ 48, 49 Moreover, Goldman et al.^28^ report that local communities adjacent to protected areas/field sites can experience increased crop raiding, livestock loss, illness, and fatalities due to closer interactions between human communities and habituated nonhuman primates. Proximity and habituation can also harm the primates through increased exposure to pathogens, increased susceptibility to hunting, poor nutrition, and/or aggressive interactions with the local humans, and this can have devastating effects on conservation efforts.^50^, ^51^, ^52^, ^53^, ^54^ Williamson and Fawcett^55^ describe how much‐needed income for local communities from increased tourism has resulted in the introduction of disease and increased stress to the vulnerable Virunga gorilla population. Red howlers habituated to human presence in tourist areas in Suriname were characterized by a greater number of botfly lesions and parasites when compared to howlers that were less habituated to human presence, and this is likely to have a negative impact on their health and long‐term survival.^56^ Focusing our long‐term work in protected and pristine areas may also skew our knowledge of how primates adjust to habitat change. For example, examining orangutans in pristine forest led to the misperception that orangutans are habitat specialists completely dependent on primary forest.^57^ The reality is far more complex, and our perception of what a given species requires to survive is incomplete. A publication bias for particular field stations and primate populations means that primatologists have provided only a narrow view into the full picture for the species.

Field sites that experience less human traffic may be better for the primates themselves because the populations experience less exposure to human researchers and potentially less attraction for the area by tourists, students, and short‐term researchers. Studies at newly founded field sites also provide new information on geographic variation and may afford a new conservation value to the area.^58^, ^59^, ^60^, ^61^ Lack of infrastructure, inconsistent funding, and inconsistent local community support may, however, make research and conservation challenging at these relatively unstudied sites. Field site managers may look for additional ways to support the area through ecotourism or field schools which can result in a lack of scientific commitment to conservation. Both long‐ and short‐term field research require evaluation and examination to document the ways our presence impacts human communities, nonhumans, and ecosystems. In Table 2, we include a set of questions and methods for evaluating these impacts and encourage researchers, field site managers, and community leaders to consider these impacts of their work on the ecology of both the human and nonhuman primate community.^43^ A cultural anthropological perspective can contribute a social definition of conservation that differs from an ecological definition in terms of its focus on a broader view of social human groups and socioecological issues. This cultural anthropological perspective can yield benefits such as increased resources due to support of local organizations, increased understanding of local systems, increased local science by community members, a better understanding of land history, and a better understanding of how to achieve conservation success.^62^

**Table 2:** Questions and methods for researcher impact assessment

Questions	Methods
What are the impacts on the local human community? Categories to consider: Economic Landscape‐crops, livestock Channeling of funding toward local organizations (schools, clinics) Primate–human interface	Survey with ethnography, ethnoprimatology, behavioral sampling
What is the land use history of the area and how does research and conservation status influence current land use?	Oral history, survey, ethnography
Does research of the field site impact intercommunity relations? Are there unequal benefits within or between communities?	Oral history, survey, ethnography
What is the local science in the area?	Literature, habitat‐country university research
What is the impact of habituation?	Behavioral sampling, survey, ethnography
What is the environmental impact of the research and researcher presence? Eco‐trash: Flagging, monitoring devices. Waste: Recycling? Trash? Water? Impact on plants and animals, and animals that are not part of the research	Inventory, monitoring
Does research, field site attract tourists?	Monitoring, record keeping
What methods are taken to mitigate negative effects?	Monitoring, record keeping

### 3.2 Is a species bias bad?

Yes and No. Long‐term knowledge of primate species in focused geographic areas has led to a greater understanding of primate variation. For example, Strier and Mendes^63^ were able to provide a detailed understanding of variation in muriqui demography, reproduction, behavioral plasticity, and group dynamics after 35 years of study. They would not have experienced the same understanding of population expansion, grouping patterns, and changes in dispersal strategies if they had stopped the work after the first 20 years of study. Long‐term work at the muriqui field site has inspired comparative studies at other sites and an increased number of conservation initiatives in Brazil. Although the muriqui field site is not one of the top publication sites identified in our literature review, it has other measures of productivity that are not evident in the English‐speaking journals represented in our sample. These outcomes include a large number of local students conducting work at the site, conservation reports to non‐English‐speaking journals/agencies or IUCN group journals, and policy outcomes (Karen Strier, personal communication). This example highlights the broader point that regional journals or non‐English journals may have an increased number of policy reports, education or workshop results, conservation initiative results, and rarer species articles (anecdotes, population status, shorter visits).

### 3.3 Theoretical and conservation implications

A comparison of primate representation in the literature through time suggests that primatologists have been focusing on a limited number of taxa since the 1950s (Table 3). Marshall et al.^61^ examined protected areas in 21 African and Asian countries to analyze where great apes are found and studied. They determined that research is highly skewed toward a very small number of sites. This can be problematic when primatologists define trends or broad behavioral or evolutionary patterns in publications and text books. Fan and Bartlett^65^ argue that a primatological bias toward great apes has resulted in a lack of attention to other species. Using Web of Science, they found 7,538 publications on six species of great apes, in contrast to 543 publications focused on 16 species of gibbons and siamangs. Due to the fact that most primates are endangered, threatened, and declining in numbers, it is critical that primatologists examine more taxa, different populations of known taxa (i.e., different field sites), and increase their conservation presence. Given that 30% of primates are critically endangered (CR), data deficient (DD), or no evaluation (NE) exists, and only 18% of published papers concerned these IUCN category of species, the window to make a difference for these species in the wild is closing. It can also be argued that there are advantages to keeping some field sites free of scientific monitoring. Strier et al.^66^ argue that researchers should consider local context and the ramifications of habituation and/or bringing attention to a given primate populations. They suggest that researchers assess both the scientific and conservation value of monitoring primate individuals.

**Table 3:** The top 10 genera represented in the 2011–2015 literature compared to representation in earlier literature

Top 10 genera 2011–2015	*N*‐publications	Top 10 genera 1931–1981^a^	*N*‐publications
*Pan*	127	*Macaca*	286
*Macaca*	125	*Papio*	229
*Alouatta*	65	*Pan*	167
*Cebus/Sapajus*	74	*Presbytis*	144
*Ateles*	45	*Cercopithecus*	121
*Papio*	42	*Gorilla*	105
*Gorilla*	33	*Alouatta*	80
*Rhinopithecus*	32	*Lemur*	70
*Pongo*	23	*Colobus*	66
*Chlorocebus*	23	*Hylobates*	48

a Includes 2,149 references from natural and seminatural settings.^64^

In 1994, Strier argued that the primate literature to date was not presenting a complete picture of variation in primate social systems, aggression, and kinship patterns. This “myth of a typical primate” not only misrepresented primate diversity, but had the potential to skew our understanding of primate (including human) evolutionary relationships.^67^ While work presented here illustrates an increase in our understanding of primate diversity since 1994, we argue that strong species and population biases still exist and have the potential to misrepresent primate patterns and influence primatological and anthropological theoretical frameworks. For example, our data suggest that the majority of work on chimpanzees has been published on populations in Tanzania and Uganda, but research on chimpanzees and bonobos from Senegal, Democratic Republic of Congo, Guinea, and Liberia^68^, ^69^, ^70^, ^71^ suggests that a more varied behavioral repertoire characterizes chimpanzees than what is shown from these popular sites. This variation should be considered when anthropologists model hominin evolution and present behavioral reconstructions of the last common ancestor between the *Homo* and *Pan* lineages.^61^^,^ 72 Past research on fancy and rare behaviors including infanticide, aggression, and “warfare” in the populations at more frequently visited sites may also skew the evolutionary importance of particular behaviors.^73^, ^74^, ^75^ Future work focusing on different populations and species will continue to provide information on primate diversity and better inform the interpretations that are the basis of ecological and evolutionary frameworks.

### 3.4 Ethics of primate fieldwork

Field primatologists and anthropologists have researched and reviewed ethical protocols since the early 2000s.^17^^,^ 29 ^,^ 44 ^,^ 46 ^,^ 52 ^,^ 54 ^,^ 76, 77, 78, 79, 80, 81, 82, 83, 84, 85, 86, 87, 88, 89 Ethical protocols involve a nested set of priorities that integrate the primate species themselves, the local human communities that live in or near primate habitats, education/outreach, maintaining primate ecosystems, and adherence to standards and guidelines required by the scientific community. Anthropologists and primatologists continue to adjust ethical protocols as they obtain new information and a better understanding of best practices in varying cultural/environmental contexts. Moreover, these researchers have learned that research and action can facilitate changes in academic culture such as new ethical requirements in the funding process, ethical statement requirements by journals, and codes of conduct at conferences and field sites.^76^^,^ 84 ^,^ 86

Field primatologists follow a number of ethical protocols both to ensure institutional compliance and to make certain their work does no harm. Publications were inconsistent in the information provided about how research followed institutional‐/habitat‐country requirements, inclusion of field site coordinates, dates of fieldwork, data collection hours, and research team member names. Not only is this bad science to exclude these details, but it makes it difficult to track the impact of the research.^23^ Field researchers should also consider what it means to be an ethical researcher and move beyond ethical checklists and compliance. We encourage the evaluation of both short‐ and long‐term ethical commitments to research sites and how researchers can foster better relationships with, and stronger long‐term commitments to, local human communities.^90^, ^91^, ^92^

### 3.5 Recommendations

Primatologists should incorporate and publish broader impacts or the direct and indirect conservation outcomes even if the work is not focused on conservation questions. Funding agencies and journals should encourage and highlight the inclusion and publication of conservation outcomes. Direct conservation outcomes include: policy outcomes, multinational involvement, conservation initiatives, and education or other outreach programs both in primate host countries and in the researcher's home country. Indirect conservation outcomes include: local involvement, local employment, long‐term stay, habitat‐country facilities/university sponsors, and student training. Major granting agencies require applicants to list broader impacts. These broader impacts should also be highlighted in primatological publications. Once broader impacts, expectations, and conservation goals and outcomes are visible and accessible, they become standard. We found that almost 50% of the publications we searched provided an ethical statement. It is important to point out that 43.2% of the publications appeared in journal issues that require an ethical statement. Therefore, only 4.8% of the remaining publications included an ethical statement where it was not required by the journal. We believe that ethical priorities should be considered during all stages of the research process from choosing a study species or field site to prioritizing how primatologists work with, and communicate with, local community members. Primatologists should also be aware of and assess research impact and include ethical priorities in publications. As role models for the future generation of primatologists and other disciplines, it is important to state our ethical priorities prominently in our publications. This means a section labeled as such in the methods even if it is not required by the journal.

## 4 CONCLUSIONS

Publications of primatological fieldwork are focused on a relatively small number of taxa at a few long‐term field stations. How well do these publications reflect the actual extent and intensity of the fieldwork being conducted? A next step is to conduct a survey of field primatologists, in order to assess whether publications are biased at the level of reviews or submissions, and what are the barriers that prevent some field primatologists from submitting or succeeding in publishing their studies in top primatological and science journals.^93^ Are these long‐term field sites characterized by a continuous research presence and support throughout the year compared to periodic return to the same site during certain months of the year? Primatologists also must consider how research on new species and new parts of a species' range can lead to knowledge on inter‐ and intraspecific variation, address gaps in primatological knowledge, and improve theoretical frameworks. Where we see increased traffic at field sites, are we measuring greater research effort or other types of ecological and human community impact, all of which should be regularly assessed using systematic methods? When primatologists examine the species that are disproportionately represented in the literature, they should carefully consider how their work contributes to novel understanding of the species and to long‐term conservation initiatives.
